# Sex and age-related differences in performance in a 24-hour ultra-cycling draft-legal event – a cross-sectional data analysis

**DOI:** 10.1186/2052-1847-6-19

**Published:** 2014-05-15

**Authors:** Lara Pozzi, Beat Knechtle, Patrizia Knechtle, Thomas Rosemann, Romuald Lepers, Christoph Alexander Rüst

**Affiliations:** 1 Institute of General Practice and Health Services Research, University of Zurich, Zurich, Switzerland; 2 Gesundheitszentrum St. Gallen, St. Gallen, Switzerland; 3 INSERM U1093, University of Burgundy, Faculty of Sport Sciences, Dijon, France

**Keywords:** Cycling, Master athletes, Sex difference, Ultra-endurance

## Abstract

**Background:**

The purpose of this study was to examine the sex and age-related differences in performance in a draft-legal ultra-cycling event.

**Methods:**

Age-related changes in performance across years were investigated in the 24-hour draft-legal cycling event held in Schötz, Switzerland, between 2000 and 2011 using multi-level regression analyses including age, repeated participation and environmental temperatures as co-variables.

**Results:**

For all finishers, the age of peak cycling performance decreased significantly (β = −0.273, p = 0.036) from 38 ± 10 to 35 ± 6 years in females but remained unchanged (β = −0.035, *p* = 0.906) at 41.0 ± 10.3 years in males. For the annual fastest females and males, the age of peak cycling performance remained unchanged at 37.3 ± 8.5 and 38.3 ± 5.4 years, respectively. For all female and male finishers, males improved significantly (β = 7.010, *p* = 0.006) the cycling distance from 497.8 ± 219.6 km to 546.7 ± 205.0 km whereas females (β = −0.085, *p* = 0.987) showed an unchanged performance of 593.7 ± 132.3 km. The mean cycling distance achieved by the male winners of 960.5 ± 51.9 km was significantly (*p <* 0.001) greater than the distance covered by the female winners with 769.7 ± 65.7 km but was not different between the sexes (*p* > 0.05). The sex difference in performance for the annual winners of 19.7 ± 7.8% remained unchanged across years (*p* > 0.05). The achieved cycling distance decreased in a curvilinear manner with advancing age. There was a significant age effect (F = 28.4, *p <* 0.0001) for cycling performance where the fastest cyclists were in age group 35–39 years.

**Conclusion:**

In this 24-h cycling draft-legal event, performance in females remained unchanged while their age of peak cycling performance decreased and performance in males improved while their age of peak cycling performance remained unchanged. The annual fastest females and males were 37.3 ± 8.5 and 38.3 ± 5.4 years old, respectively. The sex difference for the fastest finishers was ~20%. It seems that women were not able to profit from drafting to improve their ultra-cycling performance.

## Background

The most traditional endurance and ultra-endurance sports are swimming, cycling, running, and triathlon as a combination of them. In recent years, several studies reported an increased participation in ultra-endurance performances − defined as an endurance performance of six hours and longer [1] − such as ultra-running [2-4], ultra-cycling [5-8] and ultra-triathlon [9,10]. For several of these ultra-endurance events, an increased participation and an improvement in performance of master athletes older than 35 years [11] have been observed [5,12-14].

Several recent studies analysed also the influence of age and sex on triathlon performance [9,15-17]. There were also studies focusing on the influence of age and sex on running performance [18-20], however, only a few studies investigated other endurance disciplines such as swimming [21,22] or cycling [23,24]. Cycling as a non-weight-bearing activity represents an interesting model because it can be performed even in older ages [7] because of its non-technical and its non-weight-bearing character [25].

Age has been reported as an important predictor variable in ultra-endurance athletes such as ultra-marathoners [26]. An age-related decline in endurance performance is inevitable even if master athletes tend nowadays to improve their performance [12,13,18]. Endurance performance starts to decline after the age of ~55 years independently of the physical activity [25]. However, there seemed to be differences in the age-related performance decline regarding the different endurance disciplines. Ransdell *et al.* demonstrated that the age-related decline in endurance performance was exponential after the age of ~55 years for both sexes in swimming, cycling and running [25]. However, the age-related decline was less pronounced in swimming and cycling compared to running when master competitors in running, swimming and cycling were investigated [25]. For triathletes, Bernard *et al.*[27] and Lepers *et al.*[28] showed a less pronounced age-related decline in endurance performance in cycling compared to running and swimming. In contrast to Ransdell *et al.*[25], Baker and Tang [29] reported for sprint cycling that female and male master record performances decreased with advancing age in a similar manner as for swimming, rowing, weightlifting, triathlon and running. Balmer *et al.*[23] showed that cycling performance declined in an indoor 16.1-km time-trial with increasing age.

Apart from the age-related performance decline, the age at which peak performance is achieved would be of interest for endurance athletes such as cyclists to plan their career. Cycling races can be held without drafting such as cycling time trials [30], mountain bike cycling races [31], ultra-endurance cycling races [7,8] or cycling time trials in long-distance triathlons [9,15], or with drafting such as traditional road cycling races. Regarding ultra-cycling, a recent study investigating the age trends from 2001 to 2012 in a 720 km ultra-cycling race reported that the fastest female and male performance was achieved at the age of 35.9 ± 9.6 and 38.7 ± 7.8 years, respectively [7]. In a 120 km mountain bike cycling race held between 1994 and 2012, the age of the fastest athletes was lower than in a 720 km ultra-cycling race where the fastest females achieved the fastest race times at the age of 30.7 ± 5.0 years and males at the age of 27.1 ± 3.4 years [31]. These recent studies investigating performance in ultra-cycling used data from races where drafting was forbidden such as the 120 km mountain bike ultra-cycling race ‘Swiss Bike Master’ [31], the 720 km ‘Swiss Cycling Marathon’ as a qualifier for the ‘Race across America’ (RAAM) [7], and the ‘RAAM’ itself [8]. These studies showed that the participation in females was low [5,31], the performance decreased in males compared to females [31] but improved in females compared to males [7] across years, and the sex difference in performance remained unchanged [8] or decreased [7,31]. The sex difference in performance for the fastest finishers was at ~20 ± 10% in these races [5,7,8].

Drafting during cycling permits reducing average power output, oxygen consumption (VO_2_) and heart rate and therefore to improve performance [32]. Furthermore, it results in a reduction in frontal resistance and reduced energy cost at a given submaximal intensity [33]. However, no data exist about the age of peak ultra-cycling performance in a draft-legal cycling race in contrast to a non-drafting ultra-cycling performance [7]. Performance between females and males in a draft-legal ultra-cycling race might also be different as it has been shown for non-drafting ultra-cycling races [7]. Drafting might enhance female performance as it has been shown for ultra-swimming. For example, in the 34-km ‘English Channel Swim’ from Dover (Great Britain) to Calais (France) where athletes have to cover the distance alone, the fastest men were faster than the fastest women [34]. However, in the 46-km 'Manhattan Island Marathon Swim' where drafting is allowed, the best women were ~12-14% faster than the best men [35]. To date, no study investigated the age and sex of finishers in an ultra-cycling performance where drafting is allowed. If males and females were allowed to ride together with drafting, the sex difference in cycling performance might be lower compared to non-drafting conditions.

The aims of the study were to investigate the participation trends, and the sex and age-related differences in performance in ultra-cycling in a 24-hour draft-legal ultra-endurance cycling event held in Schötz, Switzerland, between 2000 and 2011. Considering existing findings in ultra-swimming, we hypothesized for a draft-legal cycling race that the sex difference in ultra-cycling performance would be lower compared to previous observations in ultra-endurance running or ultra-endurance cycling performance where drafting was not allowed.

## Methods

### Ethics

This study was approved by the Institutional Review Board of St. Gallen, Switzerland, with waiver of the requirement for informed consent given that the study involved the analysis of publicly available data.

### The race

To test our hypothesis, the age at the time of the competition and the achieved cycling distance (km) of all male and female solo riders at the 24-hours cycling race Schötz (‘24 Stunden Schötz’) were analysed from 2000 to 2011. Since 2000, the ‘24 Stunden Schötz’ has been held each year in the city of Schötz, Switzerland, in the first weekend of august. The last edition was held in 2011. Many athletes used this race to prepare for the ‘Race Across AMerica’ (RAAM). Several winners of the ‘24 Stunden Schötz’ won later the ‘RAAM’. The flat circuit extended over a distance of 9,888 m. In each lap, the athletes had to overcome 35 m difference in altitude. Solo and team riders were competing in the same field. The laps were counted electronically, drafting was allowed and solo riders were generally drafting behind team riders. The athletes had the opportunity to be assisted during the race by a personal support crew.

### Data collection and data analysis

**Figure 1:**
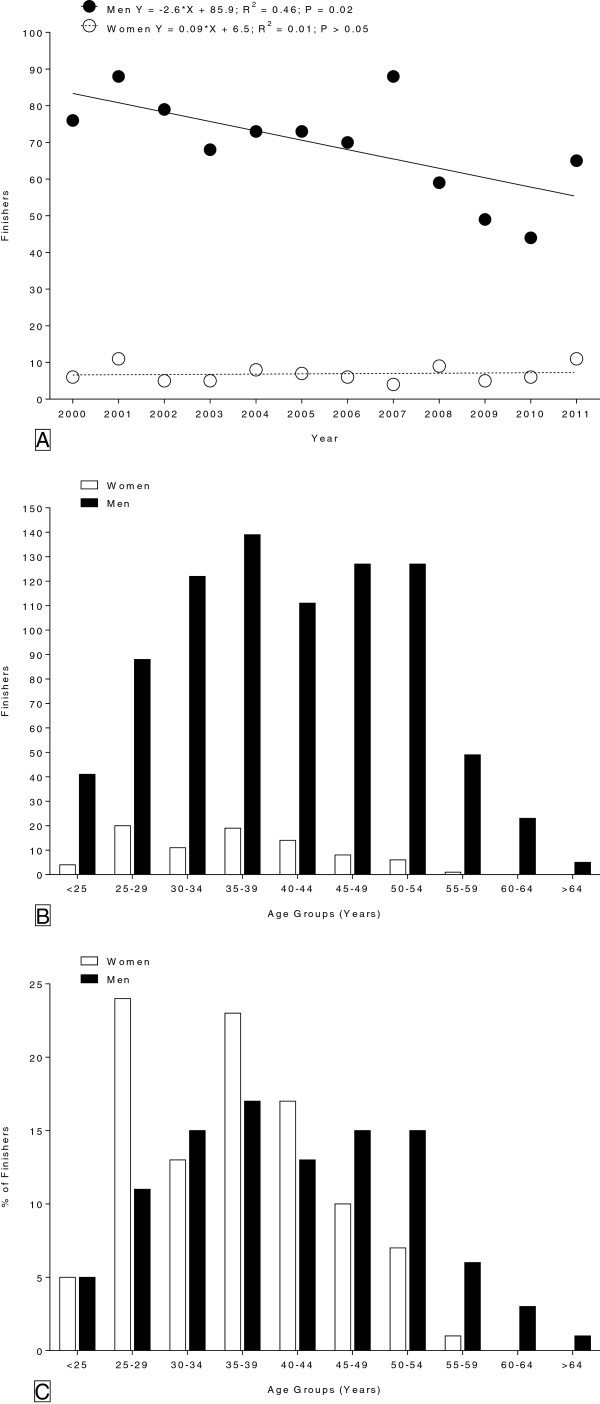
Number of female and male finishers in the ’24 Stunden Schötz’ from 2000 to 2011 (Panel A), number of female and male finishers for each age group (Panel B) and number of finishers for each age group expressed in percent of all finishers (Panel C).

The data set from this study was obtained from the race website of the ‘24 Stunden Schötz’ (http://www.24stundenrennen.ch) and from the race director for data of earlier years where the age of the athletes was missing. Between 2000 and 2011, a total of 916 finishers (*i.e.* 83 females and 833 males) completed the race. The 24-hour cycling performance was expressed in km. The age at the time of the competition and the cycling performances of all male and female competitors were analysed from 2000 to 2011. The magnitude of the sex difference was examined by calculating the percent difference between the female and male winner for each year. The effect of age on the 24-hour cycling performance was only analysed in males because the number of female finishers in the different age groups was too small for an accurate data analysis (Figure 1A). Because the age of both the annual male winners and all annual male finishers did not significantly change across the years, we pooled the data of the 12 years for the ten fastest males for each age group. The age groups distinguish the categories for each period of 5 years as follows: < 25 years, 25–29 years, 30–34 years, 35–39 years, 40–44 years, 45–49 years, 50–54 years, 55–59 years, and 60–64 years. Therefore, the best top ten performances of the athletes in nine age groups during the studied period were considered.

### Statistical analyses

Each set of data was tested for normal distribution using D’Agostino and Pearson omnibus normality test and for homogeneity of variances using Levene’s test prior to statistical analyses. Trends in participation were analysed using linear regression whereas results were tested for linearity with run’s test. Single and multi-level regression analyses were used to investigate changes in performance and age of the finishers. A hierarchical regression model avoided the impact of a cluster-effect on results where a particular athlete finished more than once. Regression analyses of performance were corrected for age of athletes to prevent a misinterpretation of the ‘age-effect’ as a ‘time-effect’ since age is an important predictor variable in ultra-endurance performance [26]. Regression models were also corrected with environmental temperatures (*i.e.* lowest and the highest temperature during every race) since environmental conditions such as extreme heat impairs endurance [30,36] and ultra-endurance performance [37,38]. Historical weather data with air temperature for this analysis were provided by “MeteoSchweiz” (http://www.meteoschweiz.admin.ch) (Table 1). The performance of the top ten athletes per age group were compared to the performance of the fastest age group using one-way analysis of variance (ANOVA) with Dunnett post-hoc analysis. Statistical analyses were performed using IBM SPSS Statistics (Version 22, IBM SPSS, Chicago, IL, USA) and GraphPad Prism (Version 6.01, GraphPad Software, La Jolla, CA, USA). Significance was accepted at *p* < 0.05 (two-sided for *t*-tests). Data in the text and figures are given as mean ± standard deviation (SD).

**Table 1:** Daily maximum (T max) and daily minimum (T min) temperatures on race days

**Year**	**T max (°C)**	**T min (°C)**
2000	20	18
2001	27	20
2002	20	17
2003	29	21
2004	26	18
2005	22	16
2006	15	10
2007	24	16
2008	25	19
2009	22	15
2010	20	16
2011	19	15

Weather data were acquired from ‘MeteoSchweiz’ (http://www.meteoschweiz.admin.ch).

## Results

### Participation trends and age groups

A total of 916 finishers (*i.e.* 83 females and 833 males) completed the race between 2000 and 2011. The annual number of finishers over the history of the event is shown in Figure 1A. The annual number of males decreased across years whereas the annual number of females remained unchanged. Between 2000 and 2011, the annual number of finishers was 70 ± 14 (range: 44–88) for males and 7 ± 2 (range: 4–11) for females, respectively. Females accounted on average for 9.2 ± 3.0% of the field over the 12-years period. The age distribution of both female and male finishers during the 12-years period is displayed in Figure 1B. Most of the male finishers were ranked in age group 35–39 years whereas most of the female finishers were in the age groups 25–29 and 35–39 years. Cyclists older than 40 years of age represented ~53% of the male and ~35% of the female finishers. Expressed in percent of all finishers, most of the male finishers were ranked in age group 25–29 years and most of the female finishers in age group 35–39 years (Figure 1C).

### The age of the cyclists

**Figure 2:**
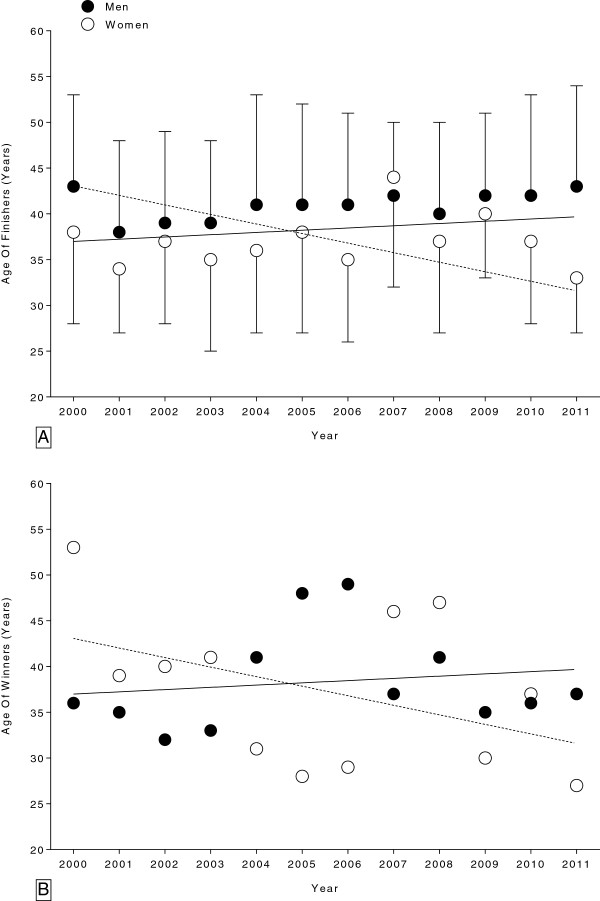
Changes in the age of all female and male finishers (Panel A) and for the annual winners (Panel B) in the ‘24 Stunden Schötz’ from 2000 to 2011.

The age of the finishers for each sex is shown in Figure 2A for all annual female and male finishers and in Figure 2B for annual female and male winners. For all female and male annual finishers, the age of peak cycling performance decreased significantly in females from 38 ± 10 years (2000) to 35 ± 6 years (2011) (Table 2). In males, however, the age of peak cycling performance remained unchanged at 41.0 ± 10.3 years (Table 2). For the annual fastest females and males, the age of peak cycling performance remained unchanged at 37.3 ± 8.5 and 38.3 ± 5.4 years, respectively.

**Table 2:** Multi-level regression analyses for change in age across years (Model 1) with correction for multiple finishes (Model 2), daily lowest air temperature (Model 3) and daily highest air temperature (Model 4) for the annual fastest and all finishers

**Model**	***β***	**SE (*β*)**	**Stand. *β***	**T**	***P***
**All male finishers**					
**1**	0.018	0.256	0.008	0.069	0.945
**2**	0.018	0.256	0.008	0.069	0.945
**3**	0.003	0.282	0.001	0.009	0.993
**4**	−0.035	0.297	−0.015	−0.119	0.906
**All female finishers**					
**1**	−0.241	0.106	0.079	2.284	0.023
**2**	−0.241	0.106	0.079	2.284	0.023
**3**	−0.205	0.094	−0.076	−2.193	0.029
**4**	−0.273	0.129	−0.073	−2.106	0.036
**Annual fastest male finishers**					
**1**	0.245	0.472	0.162	0.519	0.615
**2**	0.245	0.472	0.162	0.519	0.615
**3**	0.088	0.490	0.058	0.179	0.862
**4**	−0.191	0.464	−0.126	−0.410	0.691
**Annual fastest female finishers**					
**1**	−1.042	0.669	−0.442	−1.558	0.150
**2**	−1.042	0.669	−0.442	−1.558	0.150
**3**	−0.917	0.725	−0.389	−1.265	0.238
**4**	−0.585	0.723	−0.248	−0.809	0.439

### The performance of the cyclists

**Figure 3:**
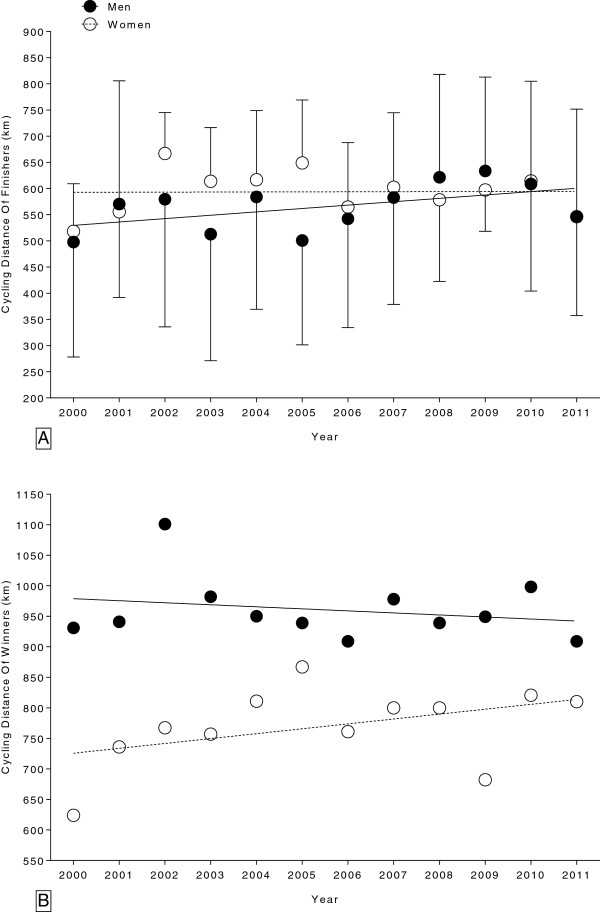
Changes in the cycling distance for all female and male finishers (Panel A) and for the annual winners (Panel B) in the ’24 Stunden Schötz’ from 2000 to 2011.

The achieved cycling distance (km) of all finishers for both sexes is presented in Figure 3A and for the annual winners in Figure 3B. Overall males improved their cycling distance significantly from 497.8 ± 219.6 km (2000) to 546.7 ± 205.0 km (2011) whereas overall females showed an unchanged performance over time of 593.7 ± 132.3 km (Table 3). The mean cycling distance covered by the male winners (960.5 ± 51.9 km) was significantly (*p <* 0.001) greater than the mean distance covered by the female winners (769.7 ± 65.7 km) (Figure 3B) but did not significantly change across the years for both sexes (Table 3).

**Table 3:** Multi-level regression analyses for change in performance across years (Model 1) with correction for multiple finishes (Model 2), age of athletes with multiple finishes (Model 3), daily lowest air temperature (Model 4) and daily highest air temperature (Model 5) for the annual fastest and all finishers

**Model**	***β***	**SE (*β*)**	**Stand. *β***	**T**	***P***
**All male finishers**					
**1**	5.614	2.222	0.087	2.527	0.012
**2**	5.614	2.222	0.087	2.527	0.012
**3**	5.452	2.229	0.085	2.446	0.015
**4**	6.424	2.317	0.100	2.773	0.006
**5**	7.010	2.538	0.109	2.762	0.006
**All female finishers**					
**1**	−0.193	4.315	−0.005	−0.045	0.964
**2**	−0.193	4.315	−0.005	−0.045	0.964
**3**	−0.176	4.335	−0.005	−0.041	0.968
**4**	0.632	4.773	0.016	0.132	0.895
**5**	−0.085	5.027	−0.002	−0.017	0.987
**Annual fastest female finishers**					
**1**	8.004	5.176	0.439	1.546	0.153
**2**	8.004	5.176	0.439	1.546	0.153
**3**	5.506	5.784	0.302	0.952	0.366
**4**	6.581	5.945	0.361	1.107	0.300
**5**	7.074	6.152	0.388	1.150	0.283
**Annual fastest male finishers**					
**1**	−3.330	4.429	−0.231	−0.752	0.469
**2**	−3.330	4.429	−0.231	−0.752	0.469
**3**	−2.098	3.993	−0.146	−0.525	0.612
**4**	−2.745	4.296	−0.191	−0.639	0.541
**5**	−3.347	4.657	−0.233	−0.719	0.493

### The sex difference in cycling performance

**Figure 4:**
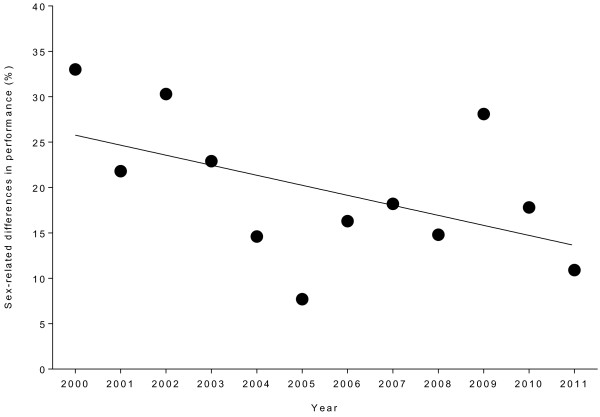
Change in the sex difference in performance for the annual winners in the ’24 Stunden Schötz’ from 2000 to 2011.

The sex difference in cycling performance for the winners during the 2000–2011 period is shown in Figure 4. The mean sex difference was 19.7 ± 7.8% (range: 7.7-33.0%) and did not significantly change across years (Table 4).

**Table 4:** Multi-level regression analyses for change in sex difference across years (Model 1) with correction for daily lowest air temperature (Model 2) and daily highest air temperature (Model 3) for the annual fastest finishers

**Model**	***β***	**SE (*β*)**	**Stand. *β***	**T**	***P***
**Annual fastest finishers**					
**1**	−1.103	0.585	−0.512	−1.887	0.089
**2**	−1.209	0.635	−0.561	−1.903	0.089
**3**	−1.106	0.695	−0.513	−1.590	0.146

### The age-related changes in male cycling performances

**Figure 5:**
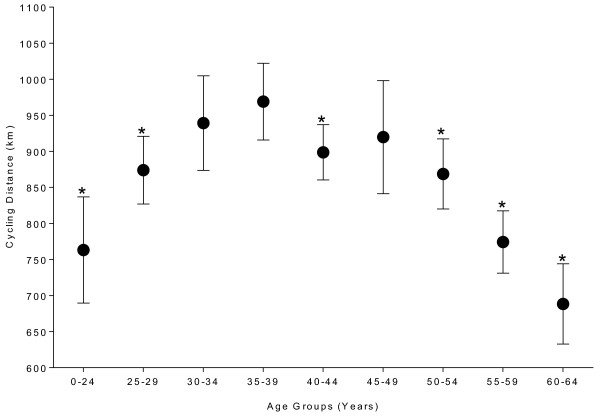
Age-related changes in male cycling performances in the ’24 Stunden Schötz’ from 2000 to 2011, * = significantly different from the fastest age group (*i.e. *35–39 years). For women, not enough data were available for statistical analyses.

The mean age-related change for the achieved cycling distance in the ten fastest males for each age group throughout the 2000–2011 period is shown in Figure 5. The achieved cycling distance decreased in a curvilinear manner with advancing age. There was a significant age effect (F = 28.4, *p <* 0.0001) for the cycling performance. The fastest cyclists were in age group 35–39 years. Cyclists younger than 34 years and cyclists older than 45 years were significantly slower than cyclists aged from 35–44 years.

## Discussion

The main findings were that females accounted for ~9% of the field, the sex difference for the annual winners was unchanged at ~20%, the performance in females remained unchanged while their age of peak cycling performance decreased and the performance in males improved while their age of peak cycling performance remained unchanged, and the annual fastest females and males were 37.3 ± 8.5 and 38.3 ± 5.4 years old, respectively.

### Participation trends regarding women and master athletes

Females accounted on average for ~9% of the field over the 12-years period. This percentage is in line with recent findings for non-drafting ultra-cycling races. By comparison, female finishers accounted for ~11% in both the ‘RAAM’ and the ‘Furnace Creek 508’ as a qualifier for the ‘RAAM’ [5]. In another qualifier for the ‘RAAM’ , the ‘Swiss Cycling Marathon’, female participation was at ~3% [5]. In other ultra-endurance sports disciplines such as running, female participation was higher. By comparison, in a 161 km ultra-marathon, female participation reached ~20% in 2004 [2]. Ultra-running seems to have a higher popularity than ultra-cycling regarding female participation.

Ultra-cycling seems to be of interest for master athletes. In the present study, cyclists older than 40 years of age represented ~53% of male finishers and ~35% of female finishers. Similar findings have been reported for non-drafting races. In two qualifiers for the ‘RAAM’ , the ‘Swiss Cycling Marathon’ and the ‘Furnace Creek 508’ , ~46% of the finishers were between 35 and 49 years of age [5]. These findings in ultra-cycling differ to findings in other ultra-endurance sports disciplines such as running. In a 161 km ultra-marathon, runners older than 40 years of age account for 65-70% of the finishers [2]. In ultra-running, Hoffman *et al.*[2] showed that the increase in the overall participation in the 161 km ultra-marathons held in North America was mostly due to the increasing number of master and female athletes . In contrast to ultra-cycling, ultra-running seems to have a higher popularity among master athletes. This could be due to the fact that only a small and a less expensive equipment is needed for ultra-running compared to ultra-cycling [6]. This makes the access to running easier in general because it is easier and less expensive to start training and racing.

### The sex difference in ultra-cycling performance

The mean cycling distance covered by the male winners was significantly greater than the distance covered by the female winners but did not significantly change across the years for both sexes. Similar findings of an unchanged sex difference in ultra-cycling performance have been reported for the ‘RAAM’ from 1982–2012 [8]. However, in the 720 km ‘Swiss Cycling Marathon’ held between 2001 and 2012 [7] and the 120 km ‘Swiss Bike Masters’ held between 1994 and 2009 [31], the sex difference in performance decreased. These disparate findings might be explained by the different lengths of the races (*i.e.* ~4,800 km in the ‘RAAM’ , 720 km in the ‘Swiss Cycling Marathon’ , 120 km in the ‘Swiss Bike Masters’ and ~600 km in the present race) and the different lengths of the investigated time periods (*i.e.* 31 years in the ‘RAAM’, 12 years in the ‘Swiss Cycling Marathon’ , 16 years in the ‘Swiss Bike Masters, and 12 years in the present race). The changes in cycling equipment (*e.g.* weight and aerodynamics of the bike) during a 12-year period might be too small to influence ultra-cycling performance.

It was hypothesized that the sex difference in ultra-cycling performance would be lower compared to previous observations in ultra-cycling. Interestingly, the mean sex difference in performance between male and female winners was ~20%. This value is close to the 23% sex difference found in the 540 km cycling split in a Triple-Iron triathlon where drafting was not allowed [9]. Also in non-drafting ultra-cycling races, the sex difference in performance for the fastest cyclist was at ~20% in the ‘RAAM’ (25.0 ± 11.9%), in the ‘Swiss Cycling Marathon’ (27.8 ± 9.4%) and in the ‘Furnace Creek 508’ (18.4 ± 13.9%) [5]. For the annual three fastest in the ‘RAAM’, the sex difference remained unchanged at 24.6 ± 3.0% [8]. For the annual three fastest in the ‘Swiss Cycling Marathon’, the sex difference decreased from 35.0 ± 9.5% in 2001 to 20.4 ± 7.7% in 2012 [7].

A special aspect regarding the ultra-endurance cycling performance in this study was drafting. Individual riders have the possibility to cycle behind the team riders and the teams were consistently changing their riders so a high average cycling speed can be sustained over the whole 24 hours for the leaders. Hausswirth and Brisswalter [32] showed that drafting in cycling permits to decrease average power output, VO_2_ and heart rate and therefore improve the performance in long-duration cycling events. Riders can cycle at a higher velocity with lower relative intensity while drafting [33]. Because drafting allows cycling at a lower VO_2_[32], drafting might be an advantage for individuals with a lower VO_2_max such as master and female athletes to achieve a better performance than in a non-drafting race. However, subjects with higher VO_2_ (*i.e.* males) can also sustain higher velocities.

Obviously, females could not profit from drafting in this 24-hour ultra-cycling draft-legal event. Limiting factors for ultra-endurance performance in females are their smaller muscle mass [39], their lower peak values inVO_2_[40] and their higher body fat [39] compared to males. Females present a disadvantage compared to males because they have a higher percent of body fat, even if they were equally well-trained [40]. In an Ironman triathlon, for example, the lower body fat was associated with a faster Ironman race time for males [41]. In long-distance triathletes, a lower percent of body fat is related to a better race performance. In female triathletes, the percent of body fat has a strong and positive relationship with total race time and both running and cycling split times. In males, however, body fat is positively related to total race time and running split time [42]. In ultra-runners, Hoffmann *et al.*[43] showed in a 161 km ultra-marathon that percent body fat was lower for finishers than for non-finishers and that percent body fat was significantly associated with race times in males.

Another explanation for the lower performance in females compared to males might be the power-to-weight ratio (PWR). Women have a lower body mass and might benefit from lower body mass in climbing. However, the main factor accounting for sex differences in peak and mean power output during cycling is the skeletal muscle mass of the lower extremities [44]. Since female athletes have a lower muscle mass [39], the power in cycling might be more limited by the skeletal muscle mass than by body mass. Additionally, laps in the ‘24 Stunden Schötz’ were flat where no climbing was needed.

In this study, we included the daily highest and the daily lowest air temperatures as co-variables to investigate a potential influence of environmental temperatures on performance since it has been reported for both runners [37,38] and cyclists [30,36] that air temperature has an influence on performance. However, performance and sex difference in performance was not influenced. This might be due to the rather moderate maximum temperatures of 23 ± 4°C (range 15–29°C) and minimum temperatures of 17 ± 3°C (range 10–21°C). Cycling performance was impaired at air temperatures of > 30°C in a 20 km time trial [36].

### The age-related decline in ultra-cycling performance

For all female and male finishers, the age of peak cycling performance decreased in females from 38 ± 10 years to 35 ± 6 years whereas in males, it remained unchanged at 41.0 ± 10.3 years. For the annual fastest females and males, the age of peak cycling performance remained unchanged at 37.3 ± 8.5 and 38.3 ± 5.4 years, respectively. The 24-hour cycling performance decreased in a curvilinear manner with advancing age and the best cycling performances were obtained in the age group 35–39 years for males. Cyclists younger than 34 years and cyclists older than 45 years were significantly slower than cyclists aged from 35–44 years.

The findings for the age of peak cycling performance were very similar to the findings in the 720 km ‘Swiss Cycling Marathon’ where the fastest males and females were at the age of 35.9 ± 9.6 and 38.7 ± 7.8 years, respectively [7]. These findings for ultra-cyclists are comparable to the findings for ultra-marathoners competing in the ‘Western State 100-Mile Endurance Run’ where the fastest race times in males were not significant different between the age groups <30 years, 30–39 years and 40–49 years [14]. Our data are also consistent with the finding that the decline in cycling performance in triathlon was not significant until the age of ~55 years [27]. However, the age-related decline in performance in 24-h road cycling appears to start later compared with a 120 km endurance mountain cycling race where a significant decline in performance was observed after the age of ~35 years [24].

The main physiological mechanism leading to a decline in performance with increasing age in endurance sports seems to be a progressive reduction of maximum oxygen uptake (VO_2_max) [45]. However, also other physiological factors such as a lower maximum strength of the lower limb muscles and a decrease in skeletal muscle mass in general [29], a slower rate of force development and transmission, and a reduction in elastic energy storage and recovery in tendons could cause a decrease in cycling performance with increasing age [46]. Cyclists younger than 34 years and cyclists older than 45 years were significantly slower than cyclists at the age of 35–44 years. This could be due to the fact that younger athletes have a lack of experience in preparation, nutrition and competing in ultra-endurance races compared to older athletes. The importance of previous experience was reported for ultra-marathoners where the personal best marathon time was the only predictor variable for race performance in a 24-hour run [47]. Also for ultra-endurance cyclists, previous personal best time was an important predictor variable [48]. Additionally, older athletes were training more and for a longer time regarding their whole life than younger athletes, who maybe just started to compete. This is in line with the finding that both personal best time and training volume were associated with race time in a mountain bike ultra-marathon [48].

### Limitations of the study

A limitation of this study is that we did not consider the training of the cyclists, their diet or their improvement in equipment. In female Ironman triathletes, training volume was related to overall race time [39] and in male ultra-endurance cyclists, both training and nutrition were related to performance [6]. Carbohydrate loading before running a marathon influences performance because it allows a runner of a given aerobic capacity and leg muscle distribution to run at greater speeds without ‘hitting the wall’, succumbing to the failure mode associated with the exhaustion of glycogen reserves and the form supplemental carbohydrate is consumed can influence the effectiveness of midrace fuelling [49]. Also, the increased cycling distance in male cyclists could be due to an improvement in equipment [50].

## Conclusion

To summarize, performance in females remained unchanged while their age of peak cycling performance decreased and performance in males improved while their age of peak cycling performance remained unchanged. For the annual winners, the mean cycling distance covered by males was significantly greater than the distance covered by females but did not significantly change across the years for both sexes. The annual fastest females and males were 37.3 ± 8.5 and 38.3 ± 5.4 years old, respectively. The sex difference for the fastest finishers was ~20% in this 24-h cycling draft-legal event and very similar to other reports from races where drafting was not allowed. It seems that women were not able to profit from the possibility of drafting to improve their ultra-cycling performance.

## Competing interests

The authors declare that they have no competing interests.

## Authors’ contributions

LP drafted the manuscript, BK and PK collected the data, and CAR and RL performed the statistical analyses, BK, CAR, TR and RL helped in drafting the manuscript. All authors read and approved the final manuscript.

## Pre-publication history

The pre-publication history for this paper can be accessed here:

http://www.biomedcentral.com/2052-1847/6/19/prepub
